# Amphibian and Avian Karyotype Evolution: Insights from Lampbrush Chromosome Studies

**DOI:** 10.3390/genes8110311

**Published:** 2017-11-08

**Authors:** Anna Zlotina, Dmitry Dedukh, Alla Krasikova

**Affiliations:** Saint-Petersburg State University, Saint-Petersburg 199034, Russia; anna-zlotina@yandex.ru (A.Z.); dmitrijdedukh@gmail.com (D.D.)

**Keywords:** lampbrush chromosomes, chromosomal evolution, amphibians, birds, karyotype, sex chromosomes

## Abstract

Amphibian and bird karyotypes typically have a complex organization, which makes them difficult for standard cytogenetic analysis. That is, amphibian chromosomes are generally large, enriched with repetitive elements, and characterized by the absence of informative banding patterns. The majority of avian karyotypes comprise a small number of relatively large macrochromosomes and numerous tiny morphologically undistinguishable microchromosomes. A good progress in investigation of amphibian and avian chromosome evolution became possible with the usage of giant lampbrush chromosomes typical for growing oocytes. Due to the giant size, peculiarities of organization and enrichment with cytological markers, lampbrush chromosomes can serve as an opportune model for comprehensive high-resolution cytogenetic and cytological investigations. Here, we review the main findings on chromosome evolution in amphibians and birds that were obtained using lampbrush chromosomes. In particular, we discuss the data on evolutionary chromosomal rearrangements, accumulation of polymorphisms, evolution of sex chromosomes as well as chromosomal changes during clonal reproduction of interspecies hybrids.

## 1. Peculiarities of Amphibian and Avian Genomes and Karyotypes

In general, amphibian and avian species are characterized by specific and complexly organized genomes. In particular, the size of amphibian genomes exhibits the greatest variety among vertebrates: in two orders, Anurans and Apoda, genome size ranges from 0.95 to 16 pg/N, while in Urodeles DNA value is extremely high and varies from 13.5 to 150 pg/N [1]. Such large genomes are enriched with repetitive sequences [2,3]. Moreover, certain Urodeles exhibit the longest intron length in comparison to other vertebrates [4]. Currently, only three amphibian genomes have been sequenced, *Xenopus tropicalis*, *Xenopus laevis* and *Nanorana parkeri* genomes, and all of them reveal quite small or average size [5,6,7]. 

Amphibian metaphase chromosomes are frequently characterized by the absence of informative banding patterns [8,9]. The number of chromosomes in diploid sets is generally low and usually does not exceed 26. Several primitive species of Urodels, Anurans and Apoda possess higher number of chromosomes sometimes including microchromosomes [10,11]. Similarities in sex chromosomes in the majority of amphibian species also complicate the karyotype analysis [12,13,14]. Furthermore, amphibians tend to hybridize and form viable and fertile interspecies hybrids that often become polyploid [15,16]. It makes investigation of amphibian genomes even more complex.

Birds have relatively small genomes with a diploid chromosome number being about 80 [17,18,19,20,21,22,23]. Typical avian karyotype comprises several pairs of relatively large macrochromosomes and numerous tiny morphologically undistinguishable microchromosomes. Notably, the microchromosomes represent a gene-dense part of the karyotype and possess about 50% of the genes [20,21,24,25]. At the same time, due to their size and DNA composition the cytogenetic and genomic data on microchromosomes remain quite limited. Currently, the most investigated avian karyotypes belong to representatives of the order Galliformes (including chicken, quails, turkey, paves, partridges, and pheasants), mainly due to their agricultural significance and importance as animal models in biological and biomedical research. Chicken (*Gallus gallus domesticus*) genome is the most comprehensively investigated avian genome. In particular, several improved drafts of chicken genome assembly have been released [21,26], the detailed description of chicken karyotype has been provided [22] and molecular fluorescent in situ hybridization (FISH) probes to individual chicken chromosomes and their particular regions have been generated [22,27,28,29,30]. It serves a reliable basis for comparative investigations of genomes and karyotypes in various bird species and representatives of other animal taxonomic groups [23,31,32,33,34,35,36,37]. At the same time, even in case of chicken, cytogenetic and genomic data on at least the microchromosomal part of the karyotype are still limited.

Taking into account the complexity of amphibian and avian karyotypes, standard cytogenetic and cytological approaches to their investigation using compact metaphase chromosomes often prove to be inappropriate. In avian and amphibian growing diplotene oocytes, chromosomes take the so-called lampbrush form. As compared with mitotic metaphase chromosomes, lampbrush chromosomes are highly decondensed, transcriptionally active and characterized by specific chromomere-loop organization [38,39,40,41]. Lampbrush chromosomes are considerably larger than their metaphase counterparts. As an example, the size of meiotic bivalents in urodeles can reach up to 700 µm as in case of a salamander *Salamandra salamandra* and a newt *Lissotriton vulgaris* (previously *Triturus vulgaris*) [38]. Lampbrush chromosomes may form loci-specific prominent loops and structures with complex morphology [38,39,40]. The nature and function of such entities mainly remain to be discovered. Nevertheless, the specific structures can serve as reliable landmarks for identification of lampbrush chromosomes and their particular regions. Based on chromomere-loop pattern, unique for each individual chromosome, as well as the distribution of marker structures, detailed cytological maps of lampbrush chromosomes can be constructed [38,42,43,44,45,46,47,48,49,50,51].

**Figure 1:**
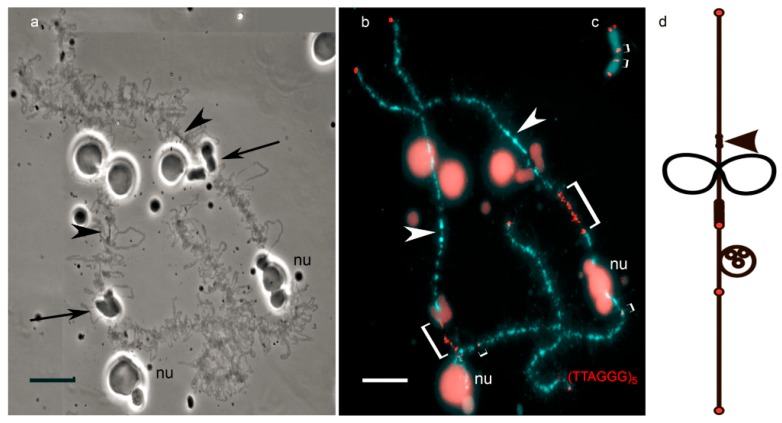
Comparison of the usage of amphibian lampbrush chromosome (**a**,**b**) and metaphase chromosome (**c**), for high-resolution fluorescent in situ hybridization (FISH)-mapping. Lampbrush chromosomes exhibit marker loops (indicated by arrows in (**a**)) and other landmarks (including nucleolus, *nu* in (**a**,**b**)), which allows to construct cytological chromosomal maps (**d**). Such maps facilitate identification of individual chromosomes and their particular regions. Mapping of interstitial telomeric sites (shown by square brackets) in: lampbrush chromosome (**b**); and metaphase chromosome (**c**). Arrowheads indicate centromeres. Chromosomes are counterstained with DAPI (4′ 6-diamidino-2-phenylindole). Scale bar = 10 µm.

**Figure 2:**
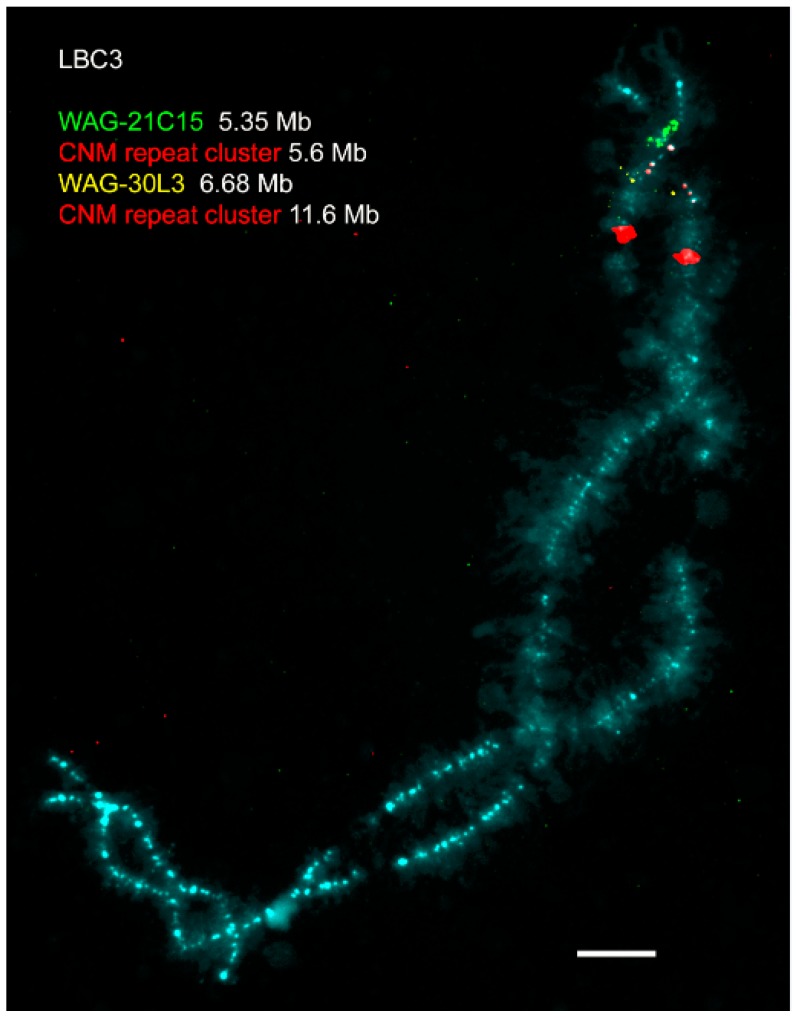
High-resolution FISH-mapping on avian lampbrush chromosomes (an example). FISH with chicken nuclear-membrane-associated repeat (CNM repeat)-specific probe (red) and bacterial artificial chromosome (BAC) clones WAG12C15 (green) and WAG30L03 (yellow) on chicken lampbrush chromosome 3. Chromosome is counterstained with DAPI. Scale bar = 10 μm. Chromosome coordinates of BACs and CNM clusters are given in megabases (Mb) according to the chicken genome assembly Gallus_gallus-5.0 (https://www.ncbi.nlm.nih.gov/genome/111) [26]. BACs were kindly provided by Richard Crooijmans and Martin Groenen (Wageningen chicken BAC library, Crooijmans et al., 2000 [29]). The data on precise genome positioning of the centromere and two CNM-repeat clusters from chicken chromosome 3 are published in Zlotina et al., 2010 [54].

Thus, due to the enormous size, peculiarities of the organization and enrichment with cytological markers, lampbrush chromosomes can serve as an opportune model for comprehensive cytogenetic and cytological investigations. Currently, the detailed protocols for preparation of lampbrush chromosomes were developed [52]. In particular, both chromosomal spreads and intact growing oocyte nuclei (germinal vesicles) can be subjected to immunofluorescent staining or different procedures of FISH (Figure 1 and Figure 2) [52,53]. 

Lampbrush chromosomes are also known in other vertebrate species including fishes and reptilians [38,55,56]. However, there is a lack of data on lampbrush chromosomes in these classes of vertebrates. In fact, studies involving reptilian lampbrush chromosomes are restricted to histological observations of ovary development [56,57] or analysis of lampbrush chromosomes in intact oocyte nuclei [58]. However, Lukina and Kupriyanova demonstrated the possibility of lampbrush chromosomes isolation for several lizard species [59,60]. Nevertheless, an adapted protocol for preparation of lampbrush chromosomes spreads, the description of chromosomal morphology and construction of detailed cytological maps are still required. Rare usage of reptilian lampbrush chromosomes is hardly explained by technical difficulties in chromosome preparation but is rather due to low interest from the cytogenetic point of view, absence of model organisms and until recently lack of reliable molecular markers (e.g., bacterial artificial chromosome (BAC)-clones and paints) [61,62,63,64]. Thus, here we focus on the main findings on chromosomal evolution that were obtained using avian and amphibian lampbrush chromosomes. 

## 2. Lampbrush Chromosomes as a Tool to Study Amphibian Chromosomal Evolution

### 2.1. Interspecies Differences

From pioneer works, amphibian lampbrush chromosomes represent an appropriate and convenient tool for analysis of amphibian karyotypes due to the abundance and variety of marker structures [38,43,51,65,66,67]. To facilitate lampbrush chromosomes identification authors constructed cytological maps with relative position of landmarks for variety of Urodeles and Anuran species [38,43,47,50,51,65,68]. Here, we focus on some of the most fascinating cases concerning karyotype evolution, accumulation of polymorphisms and sex chromosome origin provided by lampbrush chromosomes studies. 

**Figure 3:**
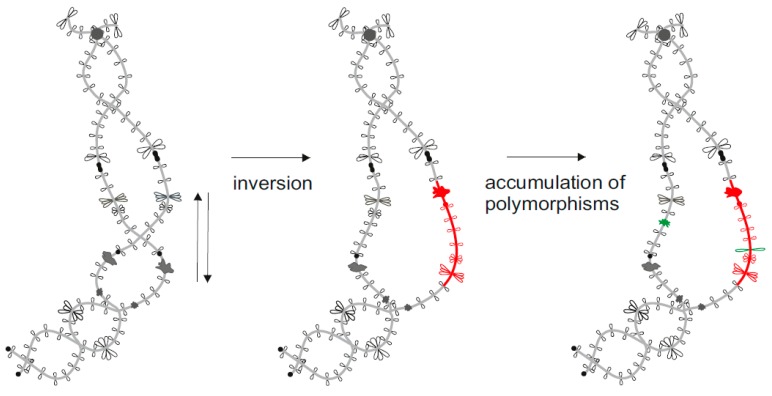
Example of lampbrush chromosome application to study chromosomal rearrangements. Inverted order of marker structures (special loops and granules) indicates the inversion of a chromosomal segment. Additional marker structures appear after accumulation of genetic polymorphisms in the absence of recombination.

Chromosomal rearrangements acquired through amphibian karyotypes evolution are less frequent compared to mammalian karyotypes [69]. Nevertheless, the level of chromosomal rearrangements in amphibian karyotypes had been underestimated for the long time and proved to be comparable with birds, reptilians and fishes [69]. Advanced salamanders and frogs species underwent quite massive karyotype rearrangements, including fusion of the ancestral chromosomes [7,69,70]. Intra- and interchromosomal rearrangements such as inversions, fusions and translocations can be detected by the analysis of orthologous lampbrush chromosomes (Figure 3) [38,51,71,72]. Based on analysis of marker structures distribution on lampbrush chromosomes, Callan [38] observed no translocations in interspecific hybrids of European newt species (genus *Triturus*). He concluded that reciprocal translocations had not occurred in chromosomes of European newts, which is in agreement with generally conservative chromosome evolution described in this genus [51,73]. 

Positions and distribution of various landmark loops on lampbrush chromosomes correlate with phylogenetic relationships between different species [38,51,74]. Thus, closely related species usually have more similar landmark patterns [51,74]. Comparison of landmarks on lampbrush chromosomes allowed to find phylogenetic relationships between different species from Urodeles and Anura, even before reliable phylogenetic markers were obtained [51,66,74]. Such comparison for three closely related Triturus species *Triturus cristatus*, *Triturus carnifer* and *Triturus karelinii* revealed similar distribution patterns of landmarks [51,75]. Lampbrush chromosomes of a sister species *Triturus marmoratus* strongly differ from lampbrush chromosomes of the species within *Triturus christatus* groups [51,75]. Nevertheless, phylogeny of more distant species is difficult to unravel via lampbrush chromosome analysis [51,66,67]. 

### 2.2. Interpopulation Differences

In addition to interspecies divergence, intraspecies genetic polymorphisms between populations can also be investigated using lampbrush chromosomes as an instrument [50,66,76,77]. Variability of lampbrush chromosome landmarks in animals from isolated populations indicates accumulation of polymorphisms (Figure 3). Such polymorphisms inevitably appear during reduced genetic flow between separate populations [78]. Thus, lampbrush chromosomes can be used as a tool for studying polymorphisms accumulation that is considered as an initial stage of allopatric speciation [78]. 

Moreover, analysis of landmarks polymorphisms allows to track distribution of species in new habitants [76]. Thorough analysis of landmarks on lampbrush chromosomes from continental and Japan populations of *Pelophylax nigromaculata* allowed to conclude that the species spread after invasion [76]. Japanese *P. nigromaculata* genetically differentiated into four groups that appeared after the termination of migration caused by geographic obstacles. Ohtani detected similarities of lampbrush marker structures between frogs from continental and certain Japanese populations [76]. This fact was considered as an evidence of introgression of genetic material in the Japanese populations after a secondary contact with the continental population of *P. nigromaculata* [76]. 

Another example of interpopulational polymorphism together with the detection of chromosomal rearrangements came from the analysis of *Rana rugosa* sex chromosomes in the lampbrush form. *R. rugosa* represents a system where sex determination type differs in distinct populations: in some populations, males are heterogametic (XX/XY) while in the other females are heterogametic (ZZ/ZW) [79]. However, in two additional populations, these frogs exhibit homomorphic sex chromosomes [79]. Analysis of marker structures on sex chromosomes in the lampbrush form revealed similarities between all different types of *R. rugosa* sex chromosomes indicating their common origin from the ancestral chromosome similar to the homomorphic one [71,72,79]. Data on distribution of chiasmata and patterns of landmarks on lampbrush chromosomes demonstrated two independent inversions resulted in the emergence of several types of sex chromosomes in this species: W/X sex chromosomes appeared after a terminal inversion, while Z/Y chromosomes appeared after a pericentric inversion of the ancestral chromosomes and subsequent deletion of approximately 10% of the chromosome [71,72,79].

### 2.3. Sex Chromosomes

Analysis of landmarks and chiasmata distribution in lampbrush chromosome allows to solve a problem of sex chromosomes identification if they are undistinguishable at metaphase. In the case of female heterogametic sex (ZW), one can detect Z and W sex chromosomes as a bivalent between two homologs with various patterns of marker loops and sometimes with a decreased level of chiasmata [12,14]. Using this approach, sex chromosomes in *Pleurodeles potreti* were identified due to a short heteromorphic region near the middle of the lampbrush bivalent 4 [14]. At the same time, in frog *Buergeria buergeri*, sex chromosomes were identified as a lampbrush bivalent with the only terminal chiasmata [80]. In another fascinating study, lampbrush chromosomes were obtained from males reversed into females after hormonal treatment [13]. Even when males’ sex is heterogametic, it is possible to apply lampbrush chromosomes to reveal differences in sex chromosomes [13]. Sex reversal experiments confirmed female heterogamety in *P. potreti*: neofemales (WW) exhibited similarity in the same region on the bivalent 4 [14]. Nevertheless, it is not always possible to observe differences between sex chromosomes during the lampbrush chromosome stage. Both females and reversed males of *Triturus* species that have XX/XY sex determination type did not exhibit any significant differences in sex chromosomes morphology [12]. 

Interesting example of sex chromosome evolution was observed after lampbrush chromosome analysis of seven species from *Triturus* genus [51,75]. In all of these species, bivalent 1 consists of a longer homolog with an extended achiasmatic region of compact loops and a shorter one with a more regular loop pattern [12,38,51,81]. Such unusual morphology is based on crucial differences in heterochromatic component of long arms between two homologous chromosomes 1: 1A and 1B [82]. Moreover, in homomorphic state (A1A1 or A2A2), all embryos usually die or exhibit severe development abnormalities [13,82]. One of the hypothesis aimed to explain the origin of this locus suggested a reciprocal translocation between two homologous autosomes in the common ancestor of species from *T. cristatus* subgenus [82,83]. Other more plausible explanation is based on the assumption that chromosome 1 is a relict sex chromosome [12]. After switching from WZ/WW to XY/XX sex determination system the majority of modern *Triturus* species are thought to eliminate Z chromosome and retain a WW chromosome pair while ancestors of species from a *T. cristatus* group probably retained ZW chromosomes which are currently known as A1 and A2 chromosomes [12,84]. 

An additional example of the application of lampbrush chromosomes in the field of karyotype evolution and sex chromosome emergence comes from investigations of frog *Leopelma hochstetteri*. Sex chromosome (W) of this species is represented by a supernumerary chromosome that highly varies in size, centromeric index and heterochromatin amount [85,86,87]. As Z chromosome was not found, authors suggested a W0/00 sex determination system for the species [85,86,88]. However, after lampbrush chromosomes analysis of frogs from one population researches did not find any supernumerary chromosomes but detected a heteromorphic lampbrush bivalent [89]. This bivalent was considered to represent a sex WZ bivalent where one homolog shared some kind of similarity in a marker loops pattern with supernumerary sex chromosomes in frogs from other populations [89]. Authors suggested that either loss of Z chromosome or its homogenization in occasional ZZW trisomy cases resulted in such a peculiar W0/00 or WZZ/ZZ sex determination type spread in other populations [86,89]. Subsequently, either translocation from W chromosome or degradation of W chromosome occurred independently in each population under Muller’s ratchet mechanism producing a variety of supernumerary W chromosomes [86,89]. In addition to supernumerary W chromosome, extra supernumerary B chromosomes varying in number, morphology and heterochromatin amount were revealed in the majority of populations [85,86]. These results indicate a higher chromosome evolution rate in *L. hochstetteri* than in other amphibians [88,90]. To explain this phenomenon, Bogart [90] proposed that rates of chromosomal evolution in amphibians might depend on the population size and animals reproductive behavior. For instance, in amphibians that form large breeding groups (Ranidae or Bufonidae) chromosomal evolution will be slower as compared to a small inbred population of neotropical frogs (such as Leopelma) with the unique breeding behavior [88,89,90].

### 2.4. Interspecies Hybrids

Amphibian species reveal a specific way of obtaining chromosomal novelties via interspecies hybridization [15,16,91]. Usually interspecies hybrids die during early development and even in case of survival cannot produce gametes [78]. After analysis of lampbrush chromosomes from oocytes of such interspecies hybrids, researchers found occasional chiasmata between orthologous chromosomes, which probably can provide chromosome separation and partial fertility [38,92]. Such hybrids represent a unique model for identification of homology regions between orthologous chromosomes after chiasmata analysis. 

Some interspecies hybrids can produce progeny via modifications of their gametogenesis including selective elimination of chromosomes originating from one parental species and/or genome duplication [91,93,94]. Such alterations of gametogenesis frequently lead to the emergence of polyploid hybrids [15,16,93,94]. Owing to the presence of species-specific landmarks, lampbrush chromosomes analysis is a useful and reliable approach to identify genomes transmitted in oocytes of di- and polyploid hybrids and to reveal genome elimination and/or duplication during hybrid gametogenesis [50,66,67,95,96,97]. Based on analysis of lampbrush chromosomes from interspecies hybrid salamanders (genus *Ambystoma*), Macgregor and Uzzell [95] inferred that genome endoreplication occurred premeiotically in germline cells allowing gynogenetic reproduction. Moreover, both elimination of one parental genome and endoreplication of another one were revealed using lampbrush chromosomes analysis in diploid and triploid hybrids from *Pelophylax esculentus* complex that reproduces via hybridogenesis [66,96]. 

Genome elimination and duplication prevent recombination events between genomes of separate species [91,93,94]. Nevertheless, occasional introgression of genetic material was observed between their genomes [98,99]. For example, having applied genomic in situ hybridization (GISH) on lampbrush chromosomes of hybrid salamanders, Bogart and coauthors [99] demonstrated extensive chromosomal exchange between genomes of parental species. According to the hypothesis of Ohno [100], such occasional recombination events result in homogenization and diploidization of parental genomes within a hybrid individual. Otherwise, the absence of recombination in the allopolyploid hybrids can subsequently lead to the independent evolution of each parental chromosomal set within the allopolyploid hybrid genome. It was clearly demonstrated for an allopolyploid frog *X. laevis* [7]. Dissimilarity in centromeric repeats between two different chromosomal sets of *X. laevis* was proven by FISH on lampbrush chromosomes. This approach allowed to identify chromosomes bearing centromeric repeat whereas in mitosis these chromosomes are of an equal size [101]. Moreover, in *X. laevis*, two genomes are characterized by different chromosomal length, chromosomal rearrangements and transposon families spread after hybridization [7].

## 3. Lampbrush Chromosomes as a Tool to Study Avian Chromosomal Evolution

Comparative molecular–cytogenetic studies involving lampbrush chromosomes proved to be helpful to reveal new evolutional changes, both inter- and intrachromosomal rearrangements, and to specify the breakpoints with high-resolution in Galliform species. In particular, it is known that some variation in chromosome number among Galliform species is mainly caused by the interchromosomal rearrangements involving ancestral chromosomes 2 and 4 [23,102,103,104,105]. As an example, using standard cytogenetic and molecular–cytogenetic approaches, it had been earlier suggested that chicken (*Gallus g. domesticus*, GGA) and turkey (*Meleagris gallopavo*, MGA) karyotypes are discriminated by two interchromosomal rearrangements with the orthologs of chicken chromosomes 2 (GGA2) and 4 (GGA4) being composed of turkey chromosomes 3 (MGA3) and 6 (MGA6), and 4 (MGA4) and 9 (MGA9), correspondingly [23]. The application of turkey chromosome painting probes for MGA3 and MGA6, as well as for MGA4 to chicken lampbrush chromosomes clearly demonstrated that the breakpoint of the interchromosomal rearrangements corresponds to the centromere of chicken chromosome 2 (GGA2) and 4 (GGA4) [105]. 

The karyotypes of chicken and Japanese quail (*Coturnix coturnix japonica*, CCO) are very similar, with the same diploid number (2*n* = 78) and high synteny conservation demonstrated repeatedly by comparative physical mapping [32,44,49,106,107], chromosome painting [31,102] and genetic linkage analysis [107,108]. At the same time, centromere position on the majority of orthologous chromosomes differs between these two species. In particular, based on the mismatch of some blocks of G-banded chromosomes [109,110,111] and the pattern of comparative FISH with cloned chicken genome sequences [32,106,107] it was suggested that pericentric inversions are responsible for the discrepancy in centromere position between chicken and quail macrochromosomes 1, 2 and 4. Dense comparative FISH-mapping of chicken BACs to chicken and quail lampbrush chromosomes confirmed the presence of the pericentric inversion between GGA2 and CCO2, revealed the inversion between GGA11 and CCO11, and allowed to narrow down their breakpoint positions [49]. At the same time, FISH on lampbrush chromosomes demonstrated the same order of molecular markers along GGA1 and CCO1 [49] as well as GGA4 and CCO4 [44] with centromeres being flanked by different genomic material in the two species. Additionally, FISH on lampbrush chromosomes revealed the inversion on chromosome 3 but, again, the difference in centromere positions between GGA3 and CCO3 could not be explained by the inversion event only [49]. The phenomenon of “centromere repositioning” or “evolutionary new centromere” (ENC) formation, where a centromere could arise during the evolution in a new chromosomal locus without any changes in the gene order, has been described repeatedly in different taxonomic groups [112,113,114,115,116,117,118]. Among birds, single cases of ENCs events have been suggested in Galliformes by comparison of orthologous chromosomes 4 of chicken and red-legged partridge [119] as well as chromosomes Z of chicken and Pekin duck [120]. The usage of elongated lampbrush chromosomes for high-resolution comparative cytogenetic analysis clearly demonstrated that “centromere repositioning” events could also take place during the divergence of chicken and Japanese quail karyotypes.

Regarding the microchromosomal part of chicken and quail karyotypes, the usage of lampbrush chromosomes [121] and synaptonemal complex spreads from pachytene nuclei [122] allowed demonstrating that the majority of Japanese quail microchromosomes are submetacentric which differs them from the chicken orthologs known to be acrocentric [121,123,124]. In particular, the dissimilarity in centromere localization was unambiguously shown by immunofluorescent detection of cohesin-enriched protein granules that serve as a reliable marker of centromeres on Galliform lampbrush chromosomes as well as by FISH-mapping of pericentromeric chicken nuclear-membrane-associated repeat (CNM) and *Bgl*II- repeat [121]. Comprehensive investigation of epigenetic chromatin state of Japanese quail microbivalents demonstrated that short arms of submetacentric microchromosomes are not completely condensed but enriched with heterochromatin protein 1 (HP1β) and repressive histone modifications, including H3K27me3 [125]. Together with the data on high-resolution comparative BAC-clone mapping on the five largest chicken microchromosomes and their quail orthologs [49], these results suggest that the discrepancy in centromere position on microchromosomes between the two species might be due to the accumulation of species-specific distinct type of heterochromatin constituting the polymorphic short arms of quail microchromosomes [125].

Thus, the usage of lampbrush chromosomes as a powerful tool for high-resolution physical mapping allows extending our knowledge of chromosomal rearrangements accompanied Galliform karyotype evolution. That is, the data obtained support the idea that the number of intrachromosomal imbalances affected highly conserved avian karyotypes seems to be higher than it could be assumed based on results of standard cytogenetic and molecular–cytogenetic analysis, which is consistent with the comprehensive molecular and in silico data on both macro- and microchromosomes rearrangements in avian genome evolution [126,127,128]. 

## 4. Conclusions

The application of classical cytological approaches, mainly based on the analysis of cytological maps and distribution of marker structures, to lampbrush chromosomes allowed to shed light on various aspects of chromosome evolution in diverse amphibian species. In comparison, avian lampbrush chromosomes possess a much lower number of cytological landmarks. In this regard, the detailed studies on avian chromosome evolution using lampbrush chromosomes became possible since modern molecular–cytogenetic techniques (FISH-mapping, chromosomal painting, etc.) became widespread. The application of lampbrush chromosomes as a tool for high-resolution cytological and cytogenetic analysis allows to obtain unique data on chromosome evolution and gives prospects for exploration of complex karyotypes, as in the case of amphibians and birds. Moreover, lampbrush chromosome analysis seems to be promising in investigation the details of chromosomal evolution in other animals including reptiles and fishes.
